# Cerebral and systemic hemodynamic effect of recurring seizures

**DOI:** 10.1038/s41598-021-01704-6

**Published:** 2021-11-15

**Authors:** Lorenzo Ferlini, Fuhong Su, Jacques Creteur, Fabio Silvio Taccone, Nicolas Gaspard

**Affiliations:** 1 Department of Neurology, Erasme Hospital, Free University of Brussels, Brussels, Belgium; 2 Department of Intensive Care, Erasme Hospital, Free University of Brussels, Brussels, Belgium

**Keywords:** Blood-brain barrier, Neuro-vascular interactions, Epilepsy

## Abstract

The increase in neuronal activity induced by a single seizure is supported by a rise in the cerebral blood flow and tissue oxygenation, a mechanism called neurovascular coupling (NVC). Whether cerebral and systemic hemodynamics are able to match neuronal activity during recurring seizures is unclear, as data from rodent models are at odds with human studies. In order to clarify this issue, we used an invasive brain and systemic monitoring to study the effects of chemically induced non-convulsive seizures in sheep. Despite an increase in neuronal activity as seizures repeat (Spearman’s ρ coefficient 0.31, *P* < 0.001), ictal variations of cerebral blood flow remained stable while it progressively increased in the inter-ictal intervals (ρ = 0.06, *P* = 0.44 and ρ = 0.22; *P* = 0.008). We also observed a progressive reduction in the inter-ictal brain tissue oxygenation (ρ =  − 0.18; *P* = 0.04), suggesting that NVC was unable to compensate for the metabolic demand of these closely repeating seizures. At the systemic level, there was a progressive reduction in blood pressure and a progressive rise in cardiac output (ρ =  − 0.22; *P* = 0.01 and ρ = 0.22; *P* = 0.01, respectively), suggesting seizure-induced autonomic dysfunction.

## Introduction

Seizures, including non-convulsive seizures (NCSz), cause a dramatic increase in cortical neuronal activity, metabolism and oxygen consumption that is normally supported by a rise in regional cerebral blood flow (CBF)^1^, owing to the mechanisms of neurovascular coupling (NVC)^2^. The effect of recurrent seizures on local and systemic hemodynamic response, and in particular the behavior of NVC to repeated solicitation, however, has not been widely investigated. It is also known that seizures affect blood–brain barrier (BBB) integrity^3–5^. A recent study in a rodent model of recurrent seizures showed that BBB permeability changes and pericytes dysfunction were associated with a progressive reduction in vessels vasodilation, probably because of a disruption of the neurovascular unit^6^. Although human BBB disruption was demonstrated in vitro^3,7^, the effects of recurrent seizures on the cerebrovascular response remain a matter a discussion since invasive brain monitoring is justifiable only in pathologic conditions which are sources of confounding factors. For example, during seizures, an abnormal neurovascular coupling, associated with a BBB dysfunction, was shown in a patient presenting a subarachnoid hemorrhage^8^ which can independently alter the NVC^9^. Moreover, this progressive reduction in vasodilation was not confirmed by human studies that, instead, showed an increase of CBF in case of non-convulsive status epilepticus^10–12^. The reasons for the discrepancy between animals and humans studies are unclear but might be related to the differences in the models (induced seizures in healthy animals vs. spontaneous seizures in brain injured patients) and to the known limits of rodent models to reproduce human pathology if compared to gyrencephalic mammalian brains^16^. Furthermore, little is known about the effect of recurrent NCSz on systemic hemodynamics, although a flattening of seizure-induced mean arterial pressure (MAP) variations has been observed^13,14^ possibly due to a progressive alteration in the function of the autonomic nervous system^15^. Finally, whether the hypothesized impairment in NVC and in systemic hemodynamic induced by recurrent seizures leads to a reduction in CBF and brain tissue oxygenation remains unknown.

In this study, we aimed to study the cerebral hemodynamic and systemic cardiovascular effects of repeated chemically-induced NCSz in a healthy ovine model, as sheep is considered a promising surrogate for modelling human brain diseases^16^ and it has already been employed as model for epilepsy^17,18^ and to study brain microcirculation^19,20^. Moreover, a sheep model offers numerous technical and theoretical advantages over small animals since it provides a unique opportunity for the transfer of techniques (neuromonitoring and neurosurgical) between animals and humans, making the results more applicable in a clinical setting^17^.

## Results

**Figure 1:**
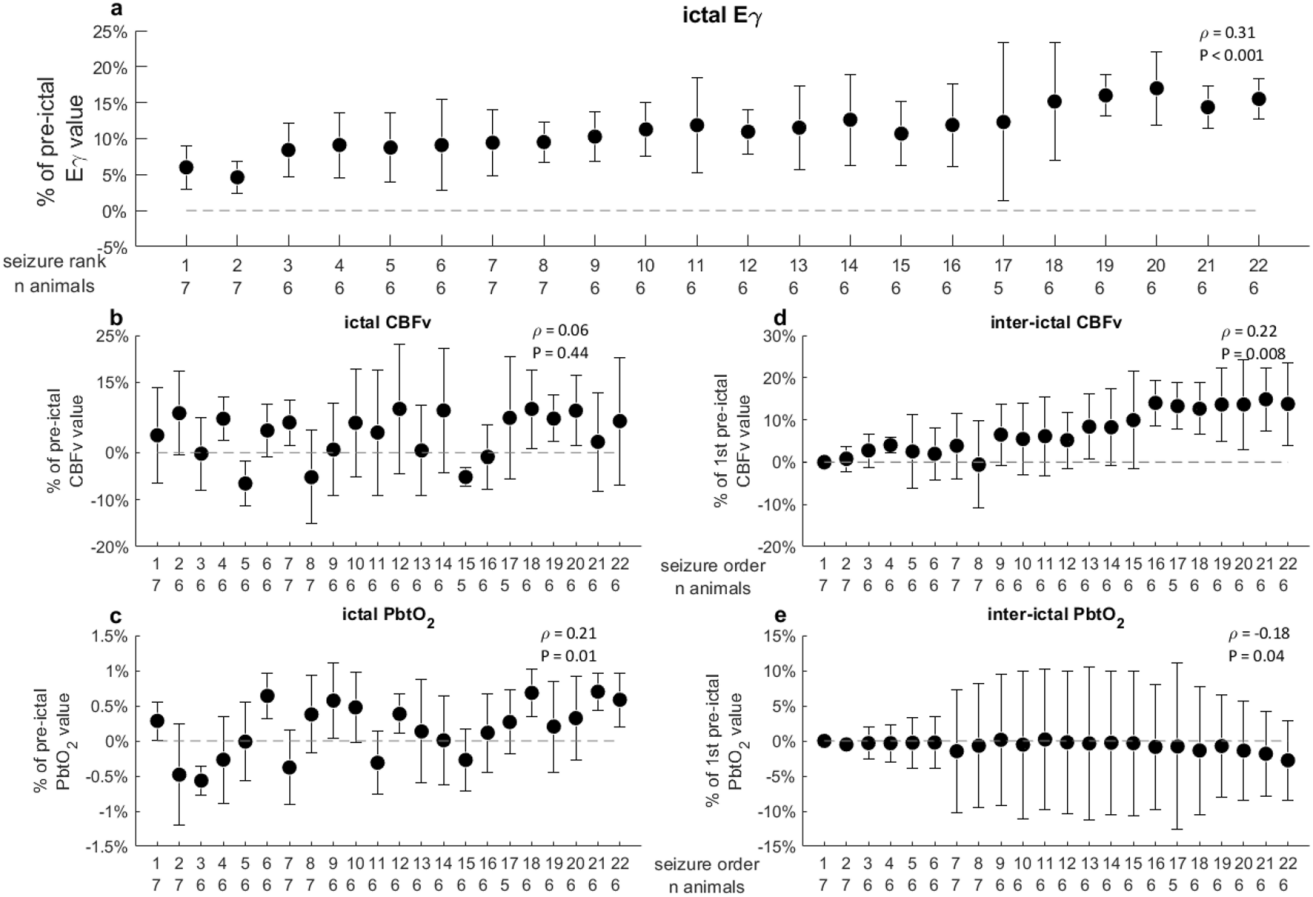
Variation of ictal and interictal cerebral parameters with seizure recurrence. *Eγ* EEG gamma envelope power, *CBFv* Cerebral blood flow velocity, *PbtO*_*2*_ Partial tissue oxygen pressure. For inter-ictal and ictal peak calculation please refer to the text. In abscissa, the seizure rank and the number of animals whose seizure was available for each rank. Data are presented as median ± median absolute deviation. The indicated Spearman’s rank-order correlation coefficient ρ and the corresponding *p* value are calculated using all available seizures. An increase in time was recorded for Eγ (ρ = 0.31, *P* < 0.001; (**a**) and ictal PbtO_2_ change (ρ = 0.21, *P* = 0.01; (**c**) whereas ictal CBFv change was not influenced by seizure recurrence (**b**). A positive statistically significant trend was noticed in the inter-ictal CBFv change as seizures recurred (ρ = 0.22, *P* < 0.01; (**d**). Conversely inter-ictal PbtO_2_ decreased (ρ =  − 0.18, *P* = 0.04; (**e**).

**Figure 2:**
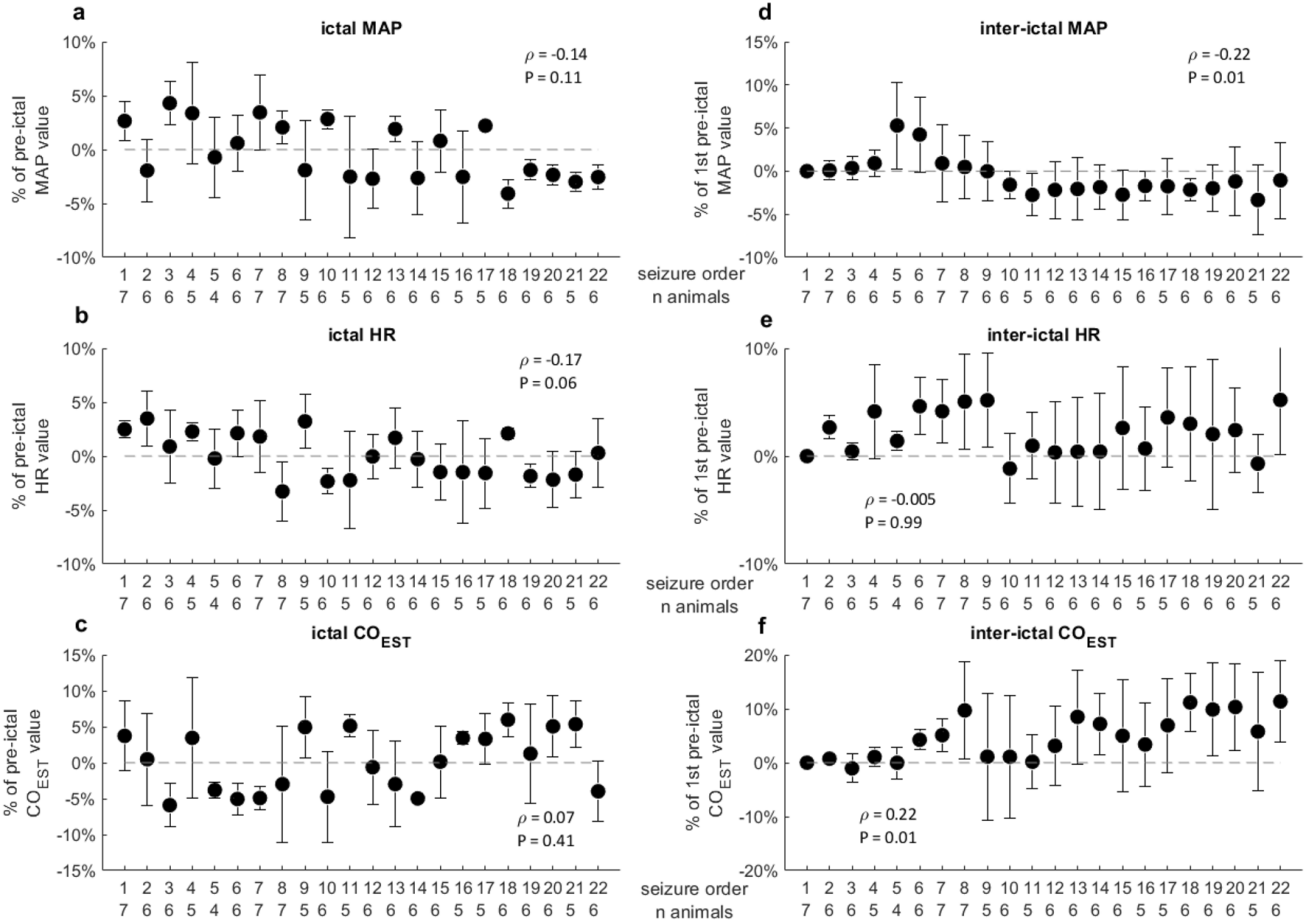
Variation of inter-ictal and ictal systemic parameters with seizure recurrence. *MAP* Mean arterial pressure, *HR* Heart rate, *CO*_*EST*_ Cardiac output estimation using the Liljestrand–Zander method. For inter-ictal and ictal peak calculation please refer to the text. In abscissa, the seizure rank and the number of animals whose seizure was available for each rank. Data are presented as median ± median absolute deviation. The indicated Spearman’s rank-order correlation coefficient ρ and the corresponding *p* value are calculated using all available seizures. A non-significant decrease in the median value of both ictal MAP (ρ =  − 0.14, *P* = 0.11; (**a**) and ictal HR (ρ =  − 0.17, *P* = 0.06; (**b**) occurred while the ictal CO_EST_ response was not influenced by seizure recurrence (**c**). The inter-ictal MAP value significantly decreased as seizures recurred (ρ =  − 0.22, *P* = 0.01; (**d**). Conversely a positive trend was observed for the inter-ictal CO_EST_ (ρ = 0.22, *P* = 0.01, (**f**).

The study included 7 sheep (median weight: 29 [range 26–35] kgs). Systemic parameters at time of penicillin application are summarized in Table 1. A median of 27 [range: 8–109] seizures were induced per animal, with a latency of 85 [42–150] min and after a cumulative dose of 1 × 10^6^ [0.5–1.6 × 10^6^] UI of penicillin (Table 1). Figures 1 and 2 show local cerebral and systemic cardiovascular effects of seizures repetition.

**Table 1:** Summary of systemic, biological parameters and information at time of penicillin application in the studied animals.

Parameter	Median [range]
CI (L/min/m^2^)	6.8 [4.1–8.5]
MAP (mmHg)	83 [79–107]
HR (bpm)	104 [95–140]
MPAP (mmHg)	13.5 [5–15]
PaCO_2_ (mmHg)	35.5 [32.2–41.5]
PaO_2_ (mmHg)	119 [111–123]
pH	7.41 [7.35–7.47]
Lactate (mmol/l)	< 1 [NA]
T (°C)	40.7 [38.4–41]
Hb (g/dl)	9.5 [8.3–9.7]
PaO2/FiO2	365 [296–498]
Weight (kg)	29 [26–35]
Interval to penicillin application (h)	6.5 [6–18]
Total penicillin dose (UI)	10 [5–16] E+05
Latency to the 1st seizure (min)	84.5 [42–150]
Number of induced seizures	27 [8–109]
Period of observation from seizure induction (h)	2.6 [1.5–5.5]
Seizure duration (s)	36.5 [17.5–59.6]
Interval between investigated seizures (min)	3.4 [1.4–29]
Cumulative MDZ dose (mg/kg)	8.2 [6.7–17.3]
Cumulative KET dose (mg/kg)	55 [44–115]

*CI* Cardiac index, *MAP* Mean arterial pressure, *HR* Heart rate, *MPAP* Mean pulmonary arterial pressure, *T* Temperature, *°C* Celsius degrees, *Hb* Hemoglobin, *MDZ* Midazolam, *KET* Ketamine, *NA* Not applicable. Data are presented as median and range.

Between seizures, periodic inter-ictal spikes were recorded with a stable median frequency of 0.8 [IQR 0.6–1.1] Hz.

### Cerebral measures

The ictal Eγ increased of a median of + 11 [7–17] % of the pre-ictal value and a statistically significant positive trend was noticed with recurring seizures (Spearman’s ρ coefficient 0.31, *P* < 0.001, Fig. 1a). An increase in the ictal CBFv was recorded in 79/133 (60%) of seizures. The median ictal variation of CBFv was + 6 [− 6 to + 15] % of the pre-ictal value, but we did not observe a significant variation with recurring seizures (ρ = 0.06; *P* = 0.44; Fig. 1b). An ictal increase in PbtO_2_ occurred in 75/135 (55%) of seizures. The median ictal variation was + 0.3 [− 0.4 to + 0.7] % and it significantly increased with recurring seizures (ρ = 0.21; *P* = 0.01; Fig. 1c). Regarding the variation of the inter-ictal measures with recurring seizures, we observed a progressive increase in the inter-ictal CBFv and a progressive decrease in the inter-ictal PbtO_2_ values (ρ = 0.22; *P* = 0.008 and − 0.18; *P* = 0.04, respectively, Fig. 1d,e).

### Systemic measures

After an initial increase until the eighth seizure, the ictal MAP and HR variations both showed a progressive decrease as seizures recurred, yielding an overall negative, albeit non-significant, trend (ρ =  − 0.14; *P* = 0.11 and − 0.17; *P* = 0.06, respectively; Fig. 2a,b). The ictal CO_EST_ variation was more heterogonous and there was no significant correlation with seizure recurrence (Fig. 2c).

The inter-ictal MAP and CO_EST_ showed an opposite behavior, with a clear negative (ρ =  − 0.22; *P* = 0.01) and a positive (ρ = 0.22; *P* = 0.01) correlation, respectively, as seizures recurred (Fig. 2d,f). The inter-ictal HR did not significantly change with recurring seizures (ρ =  − 0.005, *P* = 0.99; Fig. 2e).

## Discussion

In this experimental study, we showed that NVC is partially maintained during recurring penicillin-induced seizures in healthy animals, with stable relative ictal variations of both CBFv and PbtO_2_. This was accompanied by a progressive increase in the inter-ictal CBFv but also by a progressive decrease in the inter-ictal PbtO_2_.

Our results are thus in line with previous clinical studies that reported an increase of CBF, and a consequent rise in intracranial pressure, following repetitive NCSz^10–12^. Recurrent seizures also cause blood–brain barrier (BBB) permeability changes and vascular leakage^4,6^ leading to cerebral inflammation and brain edema, which is associated with enhanced mass effect and midline shift in brain injured patients^21^. These events might alter the neurovascular unit^22^ and potentially increase the risk of intracranial hypertension. In case of preexisting NVC alteration, as in subarachnoid hemorrhage patients, brain hypoxia might occur even during seizures^10^. As previously demonstrated^10,23^, the metabolic uncoupling during the inter-ictal phase and the seizure-induced intracranial hypertension may be a cause of secondary brain injury in critically ill patients experiencing recurrent seizures.

Prior animal studies have shown that the increased O_2_ consumption, necessary to restore basal intracellular Na^+^ and K^+^ concentrations after neuronal firing, exceeds seizure duration^24–26^, lasting up to 2 min after discharge cessation. Our results suggest that NVC cannot fully compensate for the post-ictal energy demand, resulting in progressive reduction in cerebral oxygenation, as seizures repeat. Functional hyperemia would be less efficient in restoring tissue oxygenation because of the above-mentioned seizure-induced BBB and brain extracellular milieu alterations which may worsen oxygen delivery to the tissue.

At the same time, the ictal variation in PbtO_2_ increased with recurrent seizures. It has been suggested that mitochondrial dysfunction occurs with seizure repetition^6^. Consequently, the impaired oxidative respiration could explain the reduction in O_2_ consumption and the rise in PbtO_2_ we observed as seizures repeat. Furthermore, as the lack of substrate is the rate limiting factor for oxygen consumption^28^, the inadequate inter-ictal perfusion we observed could play a role in reducing mitochondrial activity. It has to be noted that the extent of these events was not sufficient to impair neuronal function since the last seizures had a prominent EEG power and lasted longer than the early ones. As proposed by others authors, in the context of pathologic stress, auxiliary sources of energy than mitochondrial oxidative respiration, such as glycolysis, might support neuronal activity^27,29^.

Local changes at the cerebral level are further influenced by progressive hemodynamic changes at the systemic level. With recurring seizures, we observed a transient increase followed by a progressive decrease in interictal MAP variation and a progressive increase in interictal CO variation, while the ictal MAP and HR progressively decreased, albeit non significantly. As opposed to convulsive seizures and status epilepticus (SE), the systemic effects of NCSz and NCSE are not fully understood. Cardiovascular changes in epilepsy models vary accordingly to activation of different cortical (such as cingulate gyrus, insular cortex, prefrontal cortex)^30^ or subcortical (such as thalamus, hypothalamus)^31^ structures involved in cardiovascular function regulation. However, sympathetic outflow is commonly enhanced during seizures^15^. In our model, ictal-induced sympathetic stimulation resulted into an initial predominant rise in MAP associated with a chronotropic cardiac effect. Our findings are at odds with previous animal studies, which found a decrease in cardiac function responsible for a progressive reduction in ictal changes of CO and consequently MAP changes^14^. In fact, even if MAP changes show a negative tendency in both ictal and inter-ictal measures, the inter-ictal CO trend is clearly positive. Differences in species (rat vs. sheep), in the mode of administration (IV vs. in situ) and type of pro-convulsant (pentylenetetrazole vs. penicillin) may account for these discrepancies. The inter-ictal decrease in MAP and increase in CO might be explained by a differential desensitization of vascular and cardiac adrenergic receptors (AR) to persistent sympathetic activation. In different settings, α AR vascular desensitization was shown to occur in term of few hours^32^ whereas β cardiac receptors internalization might take much longer^33^. It was for instance shown that sympathetic activation during continuous NCSE only had a transient effect over peripheral vessels, as opposed to a more persistent cardiac effect^34^. This study, as well as most prior studies investigating the systemic effect of NCSE or NCSz, used IV administration of pro-convulsant agents to induce generalized seizures^14,34^.

Our study seem to suggest that seizures might progressively alter the autonomic response, in the absence of a potential direct effect of PTZ on the autonomic system. It is well known that an autonomic dysregulation occurs in epileptic patients^35^ and that it is related to the development of fatal arrhythmias and sudden death^36^.

Our study provides some leads for future clinical applications. We showed that significant changes in the cerebrovascular regulation take place after a relatively small number of seizures that might likely occur in a limited period in predisposed patients. This might suggest that, in selected cases, especially in those presenting a preexisting condition known to alter the NVC performance (i.e. traumatic brain injury^37^, ischemic stroke^38^, subarachnoid hemorrhage^10^), a real 24/7 (daytime and night time) continuous EEG (cEEG) monitoring should be provided in order to stop seizure recurrence as soon as possible and prevent their potential deleterious effects. In this sense, our results stress the importance of increasing the availability of the cEEG, in order to provide a prompt seizure treatment. In fact, cEEG is the only available tool to detect NCSz, and it is associated with a lower in-hospital mortality^39^. Unfortunately, cEEG demands high humans and technical resources and it is seldom limited to bigger health structures^39^. In order to deceive this issue, recent literature compared cEEG vs repeated routine (20 min) EEG and seemed to suggest that, even the latter was less performant in seizure detection, mortality was not influenced by recording duration^40^. Due to important methodological limitations^41^, evidences are lacking to prefer routine to continuous EEG and our results seem to suggest that efforts should be pursuit to spread cEEG use in order to maximize seizure recognition and treatment.

Our study has some limitations. First, the ictal variations in tissue oxygenation we recorded are smaller than those reported in other animals studies^42,43^. Although we positioned Clark and EEG electrodes as close as possible to each other, our measures might represent the average of PbtO_2_ variations in the focus and its surrounding tissue, which are not superimposable^42,44^, and might thus underestimate their magnitude. Second, we did not directly quantify intracranial pressure; as a consequence, we cannot confirm previous studies that found a correlation between prolonged seizure and intracranial hypertension^10–12^ and our conclusions are speculative on this point. Since animals were anesthetized, the question of whether the influence of anesthesia on NVC arises. At this moment, this effect is unclear; even if sedative drugs can alter CBF^45^, preliminary studies did not show a negative influence on NVC^46^. Whether the chosen model of epilepsy could interfere with the NVC also has to be considered. Penicillin exerts its pro-convulsant effect by inhibiting gamma-aminobutyric acid (GABA)-gated chloride ion influx^47^. GABA released from interneurons might play a role in the NVC since it has a direct effect on vascular smooth cells^48^. Blocking GABA receptors might increase neurotransmitter extracellular concentration and facilitate its interaction with vascular cells receptors. The high variability of the ictal CBFv responses is questioning. It has already been shown that the direction (increase vs decrease) of the initial vascular response to seizure depend from the distance of the recording probe from the ictal focus, since an initial decrease in the CBFv occurs in the vicinity of the seizure focus^42^. It has to be noted that an absolute increase in ictal CBFv is plausible despite the stable relative ictal variations because of an increase in the CBFv inter-ictal value, since this value was taken to calculate the ictal change. As vascular measurements were made at the brain surface, results concern mainly pial vessels and they might not be generalizable to other vascular compartments. Due to the frequently recurring seizures, it was not possible to find a suitable stationary period, as suggested by the Task Force of the European Society of Cardiology and the North American Society of Pacing and Electrophysiology^35,49^, to quantify heart rate variability and baroreflex sensitivity measurements in order to deeply analyze autonomic function. Thus, the seizure-induced autonomic dysfunction remains speculative. Finally, it has to be noticed that, in this acute setting, seizures recurrence was more frequent than what usually occurs in human patients; this precludes an entire translation of our results to clinical practice.

In conclusion, our study suggests a deleterious effect of recurrent seizures in both cerebral and systemic vascular system. The NVC is only partially maintained since it is finally unable to compensate for the metabolic demand of closely repeating seizures, leading to a reduction in brain tissue oxygenation despite a hyperemia which, at long term, might be associated with an increasing intracranial pressure that may further impair cortical perfusion. Systemic cardiovascular response changes might fail to support cerebral hemodynamics and may suggest a seizure-induced autonomic dysfunction.

## Materials and methods

### General procedure

Animal model procedures have been previously described^50^. Briefly, the Institutional Review Board for Animal Care of the Free University of Brussels (Belgium) approved all experimental procedures (approval number 675 N), which were also in compliance with ARRIVE guidelines. Care and handling of the animals were in accord with National Institutes of Health guidelines (Institute of Laboratory Animal Resources). The protocol was performed on seven anesthetized, mechanically ventilated, healthy *Ovis Aries* female sheep. Right-side craniotomy was performed using a high-speed drill (Wuhu Ruijin Medical instrument, Wuhu, China) and a 2.5 cm^2^ bone hole was opened in the frontal-parietal bone using a laminectomy tool (Aesculap-WerkeAG, Tuttlingen, Germany). The dura mater was opened with scissors and a 4-contact electrocorticography (ECOG) electrodes (Dixi Medical, Besançon, France) was slipped beneath the dura over the cortex surface of the post-central gyrus and taped to the skull. Within 1 cm from the ECOG electrode, the dura mater was subsequently punctured to insert a laser-Doppler flowmetry probe (OxyFlow 4000, Oxford Optronic, UK) for local cerebral blood flow velocity (CBFv) measurement and a Clark electrode (Licox; Integra Lifesciences, Zaventem, Belgium) for brain tissue oxygen partial pressure (PbtO2) measurement. All catheters were placed under sterile conditions at a depth of 0.5 cm into the brain parenchyma as close as possible one to each other (Supplementary Fig. S1), as previously described^50^.

### Monitoring and measurements

A continuous intravenous (IV) infusion of ketamine, morphine, and midazolam was used as general anesthesia throughout the entire experiment adjusting initial doses in order to achieve a nearly continuous background^51^. Muscular blockade was achieved using 10 μg/kg/h of rocuronium. Ventilator parameters were adjusted to maintain PaO_2_ and PaCO_2_ values in the normal (90–120 mmHg and 35–45 mmHg respectively) ranges. Mean arterial pressure (SC 9000 monitor; Siemens, Berlin, Germany), heart rate (HR), core-temperature and cardiac output (CO) (Vigilance II monitor; Edwards Lifesciences, Irvine, California, United-States) were monitored continuously. Systemic hemodynamic parameters, ECOG, PbtO_2_, and CBFv were recorded continuously and simultaneously with a sampling rate of 250 Hz (Notocord-hem, Instern Company, France). Measurements of mean pulmonary arterial pressure were collected every 1.5 h. Cardiac index (CI) was calculated using standard formulas^52^; the body surface area was estimated from Mitchell’s sheep-specific formula^53^.

### Experimental protocol

After surgical procedures, the animal was allowed to stabilize. Seizures were induced by topical application of penicillin G (Kela Pharma, Sint-Niklass, Belgium) on the cerebral cortex beneath the ECOG electrode. 10^5^ penicillin UI, diluted in 0.1 mL of CNS perfusion fluid (Perfusion Fluid CNS, CMA Microdialysis AB, Sweden), were applied every 15 min until seizures were induced. After data collection, animals were euthanized using IV high dose of potassium chloride (40 mEq).

### Data analysis

**Figure 3:**
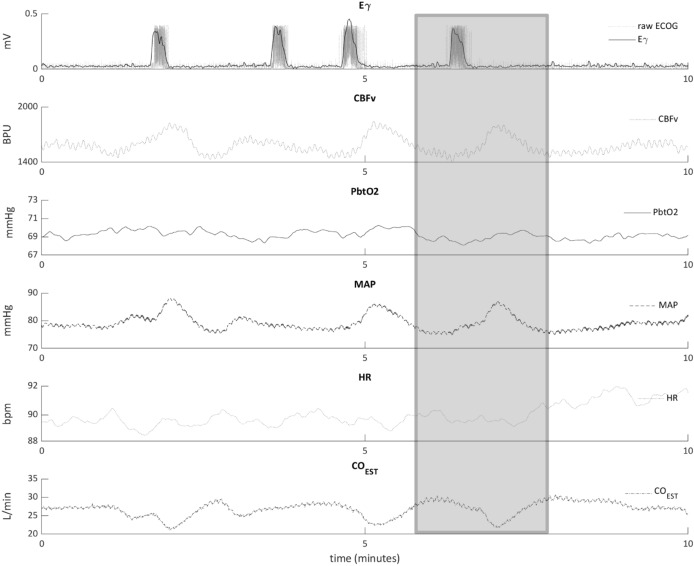
Multimodal cerebral and systemic recording in an animal with penicillin-induced seizures. *Eγ* EEG gamma envelope power, *CBFv* Cerebral blood flow velocity, *PbtO*_*2*_ Partial tissue oxygen pressure, *MAP* Mean arterial pressure, *HR* Heart rate, *CO*_*EST*_ Cardiac output estimation using the Liljestrand–Zander method. Time = 0 corresponds to the last penicillin application before seizure occurrence. Peaks in the gamma envelope of the ECOG signal corresponds to induced seizures on electrocorticography (Eγ—top row). Seizures are associated with time-locked variations in local CBFv (second row), PbtO2 (third row) and systemic parameters (MAP—forth row, HR—fifth row, and CO_EST_—last row). The epoch around a seizure is presented as example (grey rectangle); for more details, please refer to the text.

All analyses were performed offline, using built-in and custom functions in Matlab (The MathWorks, Natick, MA, USA). Physiologically implausible measures and outliers, defined according to Chauvenet’s criteria^54^, were removed. Seizure onsets were defined as rhythmic discharges with a frequency ≥ 3 Hz, according to consensus criteria^51^; seizure offsets corresponded to post-ictal ECOG signal attenuation or when discharge frequency dropped below 3 Hz. For each detected seizure, ECOG epochs from 30 s before to 60 s after seizure onset were selected (Fig. 3); if the inter-ictal interval between two consecutive seizures was shorter than 60 s, both seizures were discarded. The envelope of the gamma frequency band (30–50 Hz) (Eγ) was extracted from the ECOG signal using wavelet transform spectral density estimate (*cwt*, *icwt* and *envelope* functions in Matlab); this frequency band was chosen because it best reflects neurons’ activity^55^. Physiological variables (MAP, HR, CBFv, PbtO_2_ and CO) were simultaneously recorded; since the sampling frequency of the CO monitor was too low to identify rapid variations, the Liljestrand-Zander method^56^ was employed to estimate CO from the pulse waveform (CO_EST_). The CBFv signal was high-pass filtered with a cut-off frequency of 0.25 Hz. Since the number of seizures varied between animals, data analysis was limited to the first 22 seizures per animal in order to maximize the available variables. Of note, since animals were curarized, seizures were non-convulsive.

### Ictal versus pre-ictal variations

For each seizure of each animal, all variables were expressed as percentage variations from the baseline mean, calculated on the pre-ictal 15 s epoch, as previously described^10^. The peak values of the normalized ictal measures for each seizure were pooled together across animals and used for further statistical analysis.

### Variations of pre-ictal values

For each seizure of each animal, pre-ictal values were also expressed as percentage variations of the pre-ictal mean of the first seizure; each animal was used as its own baseline reference. These normalized pre-ictal values for each seizure were pooled together across all animals and used for further statistical analysis.

### Statistical analysis

Statistical analyses were performed using Matlab (MathWorks). A *p* value < 0.05 was considered statistically significant. Data are presented as median ± median absolute deviation or median [range]. The Spearman’s rank-order correlation coefficient was used to measure the strength of the association between all available repeating seizures and variations of local (Eγ, CBG, PbtO_2_) and systemic (MAP, HR, CO_EST_) parameters.

To ensure that measured parameters were stable over time, they were recorded in four control animals in which seizures were not induced. Results are presented in Supplementary Fig. S2.

### Ethical publication statement

We confirm that we have read the Journal’s position on issues involved in ethical publication and affirm that this report is consistent with those guidelines.

## Supplementary Information

Supplementary Information.
