# Supplementary material for: Cerebral and systemic hemodynamic effect of recurring seizures
Source: Sci Rep. 2021 Nov 15;11:22209. doi: 10.1038/s41598-021-01704-6 (PMC8593180; doi:10.1038/s41598-021-01704-6)
Supplement: Supplementary file 1 — Supplementary Information. [file 41598_2021_1704_MOESM1_ESM.docx]

**SUPPLEMENTARY FIGURE LEGENDS**


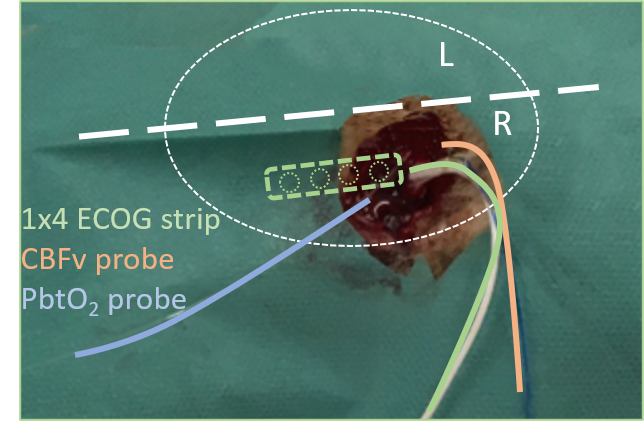
**Figure S1.** **Schema of probe positioning (viewed from above the skull of the animal in prone position).** A unique hole of craniotomy in the right frontal-parietal bone is visible. A 4-contact electrocorticography electrode (ECOG strip, in transparency), a Clark electrode for brain tissue oxygen partial pressure (PbtO2 probe) measurement and a laser-Doppler flowmetry probe for local cerebral blood flow velocity (CBFv probe) measurement were placed under sterile conditions at a depth of 0.5 cm into the right-side brain parenchyma as close as possible one to each other.


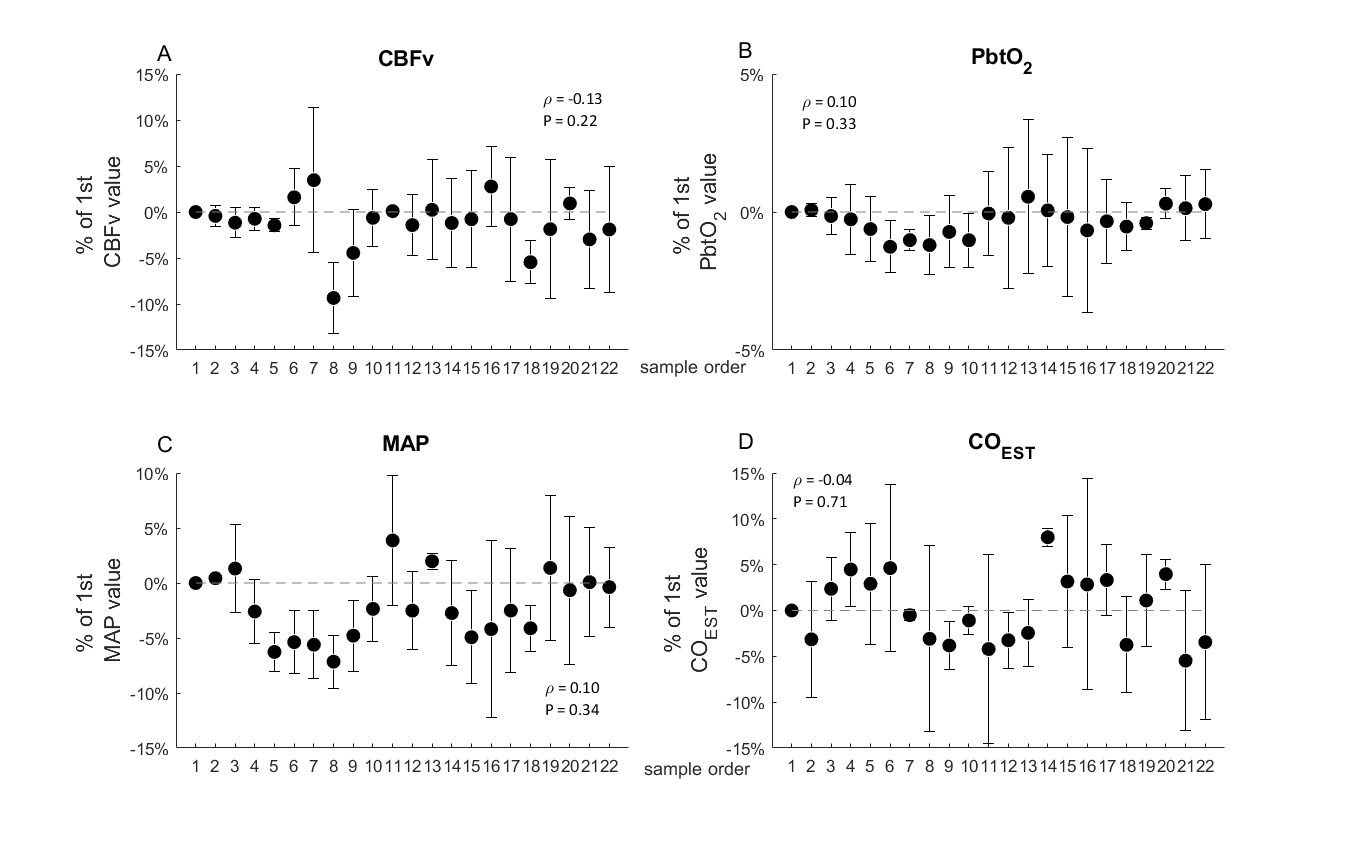
**Figure S2. Variation of cerebral and systemic parameters in control animals without seizure induction.** CBFv: cerebral blood flow velocity, PbtO2: partial tissue oxygen pressure, MAP: mean arterial pressure, CO_EST_: cardiac output estimation using the Liljestrand-Zander method. Cerebral (panels A & B) and systemic (panels C & D) parameters were analyzed in four control animals were seizures were not induced. Parameters were recorded starting after the corresponding median epoch to penicillin application in epileptic animals for their median interval of observation (i.e. 6.5h after the beginning of the experiment, for 2.6 h of observation; please refer to Table 1 in the text). This interval was divided into 22 sections, corresponding to the 22 considered seizures in epileptic animals, and, for each subsection, the first 15 seconds of cerebral and systemic parameters were analyzed as reported in the method section of the text. Data are presented as median ± median absolute deviation. The indicated Spearman’s rank-order correlation coefficient ρ and the corresponding P value are calculated using all available values. Despite a slight variability, no parameters present a significant trend in time.
